# Opioids for treating refractory dyspnea in patients with heart failure: A protocol for systematic review and meta-analysis: Retraction

**DOI:** 10.1097/MD.0000000000034401

**Published:** 2023-07-07

The article “Opioids for treating refractory dyspnea in patients with heart failure: A protocol for systematic review and meta-analysis”,^1^ which appeared in Volume 101, Issue 47 of *Medicine®* is being retracted due to plagiarism of an existing PROSPERO study protocol record,^2^ falsification of authorship, and falsification of the License to Publish agreement.
